# POSTOPERATIVE CHANGES IN INTESTINAL MICROBIOTA AND USE OF PROBIOTICS IN ROUX-EN-Y GASTRIC BYPASS AND SLEEVE VERTICAL GASTRECTOMY: AN INTEGRATIVE REVIEW

**DOI:** 10.1590/0102-672020180001e1400

**Published:** 2018-12-06

**Authors:** Nathalia Ramori Farinha WAGNER, Marilia Rizzon ZAPAROLLI, Magda Rosa Ramos CRUZ, Maria Eliana Madalozzo SCHIEFERDECKER, Antônio Carlos Ligocki CAMPOS

**Affiliations:** 1 Postgraduate Program in Food and Nutrition; 2 Postgraduate Program in Clinical Surgery, Federal University of Paraná, Curitiba, PR, Brazil

**Keywords:** Bariatric surgery, Cirurgia bariátrica, Probióticos, Microbiota, Microbioma gastrointestinal

## Abstract

**Introduction:**

Studies suggest that weight loss induced by bariatric surgery and the remission of some comorbidities may be related to changes in the microbiota profile of individuals undergoing this procedure. In addition, there is evidence that manipulation of the intestinal microbiota may prove to be a therapeutic approach against obesity and metabolic diseases.

**Objective:**

To verify the changes that occur in the intestinal microbiota of patients undergoing bariatric surgery, and the impact of the usage of probiotics in this population.

**Methods:**

Articles published between 2007 and 2017 were searched in Medline, Lilacs and Pubmed with the headings: bariatric surgery, microbiota, microbiome and probiotics, in Portuguese, English and Spanish. Of the 166 articles found, only those studies in adults subjected to either Roux-en-Y gastric bypass or sleeve vertical gastrectomy published in original articles were enrolled. In the end, five studies on the change of intestinal microbiota composition, four on the indirect effects of those changes and three on the probiotics administration on this population were enrolled and characterized.

**Conclusion:**

Bariatric surgery provides changes in intestinal microbiota, with a relative increase of the Bacteroidetes and Proteobacteria phyla and reduction of Firmicutes. This is possibly due to changes in the gastro-intestinal flux, coupled with a reduction in acidity, in addition to changes in eating habits. The usage of probiotics seems to reduce the gastro-intestinal symptoms in the post-surgery, favor the increase of vitamin B12 synthesis, as well as potentiate weight loss.

## RESUMO

**Introdução:**

Estudos sugerem que a perda de peso induzida pela cirurgia bariátrica e a remissão de algumas comorbidades podem estar relacionadas às mudanças no perfil da microbiota dos indivíduos submetidos a este procedimento. Além disso, há indícios de que a manipulação da microbiota intestinal pode vir a ser abordagem terapêutica contra a obesidade e doenças metabólicas.

**Objetivo:**

Verificar as mudanças que ocorrem na microbiota intestinal de pacientes submetidos à cirurgia bariátrica, e o impacto do uso dos probióticos nessa população.

**Métodos:**

Foi realizada a busca de artigos publicados entre os anos de 2007 e 2017 nas bases de dados Medline, Lilacs e PubMed com os descritores: cirurgia bariátrica, microbiota, microbioma e probióticos em português, inglês e espanhol. Dos 166 artigos encontrados, foram selecionados apenas os estudos realizados em adultos, submetidos ao bypass gástrico em Y-de-Roux ou gastrectomia vertical sleeve publicados em artigos originais. Ao final, foram selecionados e categorizados cinco estudos sobre a mudança na composição da microbiota intestinal, quatro sobre os efeitos indiretos dessas mudanças e três sobre a administração de probióticos nessa população.

**Conclusão:**

A cirurgia bariátrica proporciona mudanças na microbiota intestinal com aumento relativo dos filos Bacteroidetes e Proteobactéria e redução de Firmicutes. Isso se deve, possivelmente, às alterações no trânsito gastrointestinal com redução da acidez intestinal além de modificação dos hábitos alimentares. O uso de probióticos parece reduzir os sintomas gastrointestinais no pós-operatório, favorecer o aumento de síntese de vitamina B12 e potencializar a perda de peso.

## INTRODUCTION

Obesity is defined as an abnormal accumulation or an excess of body fat, which may reach a health-impairing degree. Its etiology is multifactorial and complex, resulting from an interaction of genes, environment, lifestyle and emotional factors^5^. When obesity is severe, bariatric surgery is the treatment with the most consistent results in excess weight loss, remission of comorbidities and improving life quality^1^. Roux-en-Y gastric bypass (RYGB) and sleeve vertical gastrectomy (SL) are the two most used procedures for such cases^1^ ^,^ ^20^. The first consists of a technique that couples the reduction of gastric volume and a detour of the proximal intestine, while the latter is a restrictive technique in which 80% of the greater curvature of the stomach is ressected^1^. 

During the last couple decades, a lot has been learned on the physiological mechanisms, as well as the neuro-hormonal circuits and their functions in the control of the body composition, of the genes and of the mechanisms that determine the susceptibility to obesity^9^ ^,^ ^12^. With the advancement of human gene sequencing, it has become possible to study the variety of microorganism communities present in the intestinal ecosystem. As such, some evidence has emerged that links some phyla of distinct bacteria to metabolic diseases. Recently, intestinal dysbiosis has been considered an additional factor for the development of obesity and type II diabetes melittus^15^ ^,^ ^27^.

The intestinal flora may be described as a microbiome (a collection of micro-organisms in an environment) or a microbiota (the micro-organisms themselves)². It is estimated that in the gastrointestinal tract there are approximately 10^14^ micro-organisms, composed by more than a thousand distinct kinds of species and more than three million genes, compared to approximately 30.000 in human genome, showing a co-evolutionary pathway^17^. This microbiota has been shown to interact with the host in a symbiotic way, modulating inflammation and the immune system; acting in the biotransformation of xenobiotics and in the absorption of micronutrients; synthetizing vitamins, enzymes and proteins used by the host; fermenting energetic substrates; providing resistance to pathogens; and changing amount of available energy in the diet^15^ ^,^ ^17^ ^,^ ^22^ ^,^ ^23^

Studies suggest that weight loss induced by bariatric surgery and the remission of some comorbidities, such as diabetes mellitus type II, may be related to changes in microbiota of individuals subjected to this procedure. However, the impact of the surgery in the composition and function of intestinal microbiota is still unclear^14^.

Considering the notion that there are differences in the intestinal colonization of eutrophic and obese individuals, studies have been suggesting the use of probiotics - living micro-organisms that, when administered in adequate amounts, may bestow health benefits to their host^15^ - for body weight management. However limited are the human studies in this field, there is evidence that the manipulation of intestinal microbiota may become a therapeutic approach in the treatment of obesity and metabolic diseases^16^ ^,^ ^17^.

Thus, the goal of this research was to verify the changes that occur in the intestinal microbiota of patients submitted to bariatric surgery, and the impact of the usage of probiotics in this population.

## METHODS

The present article is an integrative review of literature, carried out with the elaboration of the guiding question, the establishment of the criteria for article selection, preparation of the instrument for data collection, presentation of the results and interpretation of the information collected.

The guiding questions of this study were: “What are the changes in intestinal microbiota of individuals submitted to bariatric surgery?” and “What are the effects of probiotics in health and life quality of the patients following bariatric surgery?”.

For the search and selection of papers, the databases Pubmed, Medline and Lilacs were used, with “probiotics”, “microbiota” and “microbiome” as the Descriptors in Health Science (DHS). Those descriptors were coupled to the term “Bariatric Surgery”.

Original articles were enrolled in the study, published in between the years of 2007 and 2017, which are fully available online. Their subject was the changes in intestinal microbiota following bariatric surgery and the effects of probiotics in health and quality of life of this population. Only studies in adults subjected to either RYGB or SL were enrolled, published in English, Portuguese and Spanish. Papers whose content did not address the subjects under study, theses, dissertations, literature reviews and case studies were excluded from the study.

The studies were catalogued by specific instrument, with items such as: reference (author name and year of publication), study objectives, sample, time elapsed following surgery and main results found. For the tabulation of probiotic studies, in addition to the aforementioned items, the following were added: type of study and period of data collection, with the description of the strains used in the column referring to the characterization of the sample. 

From the results found, the studies were categorized into three tables. The ones that presented changes in the composition of the intestinal microbiota of subjects undergoing bariatric surgery were presented in Table 1. Others categorized in Table 2 indicated the changes in the plasma dosages of products derived from the metabolism of intestinal bacteria, indicating changes in the microbiota after surgery. Finally, Table 3 shows the studies that analyzed the impact of the use of probiotics on the health of individuals undergoing bariatric surgery.

**TABLE 1:** Analysis of the changes in the composition of the intestinal microbiota of individuals subjected to bariatric surgery.

Authors, year	Objective	Sample	Time of surgery	Main results found
Furet *et al.*, 2010	To analyze the impact of RYGB on the changes in intestinal microbiota and to examine links with adaptations associated to this procedure	CONTROL GROUP (CG): 13 lean individuals (women) OBESE GROUP (OG): 30 obese subjects submitted to RYGB (27 women and 3 men)	Pre-surgery, 3 to 6 months following surgery	Group Bacteroides/*Prevotella* was lower in OG before surgery than in CG and increased at 3 months of surgery; was negatively correlated with corpulence, and the relationship was highly dependent on food intake. *Escherichia coli* increased at 3 months of surgery and was inversely correlated with fat mass and leptin level, regardless of dietary intake. *Lactobacillus / Leuconostoc / Pediococcus* and *Bifidobacterium* group decreased at 3 months of surgery. *Faecalibacterium prausnitzii* was lower in subjects with diabetes and negatively associated with inflammatory markers before and after surgery, regardless of changes in food intake.
Zhang, 2009	To identify specific microbial lineages that may play important roles in the development of obesity and also determine if the presence or abundance of these microorganisms changes after successful RYGB	9 subjects: 3 eutrophic, 3 morbidly obese and 3 after RYGB	>6 months	Dominance of the phylum Firmicutes in eutrophic and obese individuals and significantly lower in those who underwent RYGB. These had a marked increase in the relative abundance of Gammaproteobacteria and proportionally less Clostridia when compared to the other groups.
Tremaroli, 2015	To investigate the long-term effects of bariatric surgery on the microbiota of patients submitted to RYGB and Vertical Band and compare weight loss and fat mass	21 women: 7 RYGB and 7 VBG + 7 women with severe obesity	9.4 years	Significant difference in the microbiota between women of the RYGB and obese group: Gammaproteobacteria was higher while 3 species of the Firmicutes phylum (*Clostridium difficile, C. hiranonis* and *Gemella sanguinis*) were lower in the women submitted to the RYGB. As well as the presence of Proteobacteria was higher in the RYGB group than in the obese group. Increased levels of TMAO in the RYGB group.
Kong, 2013	To determine the impact of RYGB on the changes in the intestinal microbiota and the potential associations with changes in gene expression in WAT	30 obese women (7 diabetic and 23 non-diabetic) submitted to RYGB and evaluated before and after surgery	Pre-surgery, 3 and 6 months following surgery	The richness of the intestinal microbiota increased following RYGB; 37% of the increased bacteria belonged to Proteobacteria. The associations between intestinal microbiota composition and WAT gene expression increased following RYGB. The profile of bacteria before surgery changed significantly at 3 and 6 months of RYGB, without significant differences between the 3rd and 6th month. Bacteria belonging to the phylum Firmicutes *(Lactobacillus, Dorea* and *Blautia*) and *Bifidobacterium* (from the phylum Actinobacteria) decreased and those belonging to the phylum Bacteroidetes (*Bacteroides* and *Alistipes*) increased significantly after RYGB. As well as the genus *Escherichia*, belonging to the phylum Proteobacteria, also increased after the surgery
Graessler, 2012	To characterize intra-individual changes in fecal microbiota composition of morbidly obese individuals by metagenomic sequencing before and after 3 months of RYGB.	6 subjects with morbid obesity (5 with type 2 DM) submitted to RYGB	Pre-surgery, 3 months following surgery	Significant changes in the intestinal microbiota were observed in 22 species, 11 genera of bacteria. Proteobacteria, Verrucomicrobia and Fusobacteria had increased participation of the microbiota after surgery, and the phyla Actinobacteria, Cyanobacteria, Firmicutes and Bacteroidetes decreased. However, the Bacteroidetes/ Firmicutes ratio showed an apparent increase. The genera *Faecalibacterium* and *Eubacterium* decreased and *Akkermansia* and *Escherichia* increased in the postoperative period.

RYGB=Roux-en-Y Bypass; TMAO=Thrimethylamine N-oxide; WAT= white adipose tissue; VBG=vertical band gastroplasty; DM:=Diabetes Mellitus

**TABLE 2:** Analysis of the indirect effects of changes in the composition of the intestinal microbiota in individuals submitted to bariatric surgery

Authors, year	Objectives	Samples	Time of surgery	Main results found
Sarosiek *et al.*, 2016	Provide information regarding the mechanism by which the bariatric surgical procedures lead to weight loss and a reduction or resolution of diabetes.	Total of 15 patients subjected to either SL or Bypass, with or without Diabetes Type II	Pre-surgery, 28 days following surgery	Large increase of histidine after bariatric surgery possibly derived from altered composition of intestinal flora
Clemente-Postigo, 2015	To analyze the effects of 2 surgical techniques (SL and bilio-pancreatic deviation) on plasma levels of LPS and LPS binding protein	50 obese individuals subjected to bariatric surgery, among these 24 subjected to SL, between 2011 and 2013	Pre-surgery, 15 and 90 days following surgery	The individuals subjected to SL have shown significant reduction of LPS by 90 days following surgery. The levels of LPS binding protein has been reduced 90 days following surgery in the normoglicemic and pre-diabetic/diabetic groups.
Modesitt, 2015	To determine baseline endometrial histology in morbidly obese women undergoing bariatric surgery and to evaluate the impact of surgical intervention on serum metabolic parameters, quality of life and body weight.	71 women: 43 subjected to RYGB and 17 to SL	Pre-surgery, 6 and 12 months following surgery	Significant disturbances in Tryptophan, Phenylalanine and heme metabolism suggest changes in intestinal microbiota and decreased inflammation.
Troseid, 2016	To investigate the potential impact of obesity, of lifestyle intervention and of bariatric surgery on the pro-atherogenic metabolic TMAO as well as its microbiota-dependent intermediate gamma-butyroatine and its dietary precursors choline and carnitine in morbidly obese subjects.	34 obese individuals subjected to RYGB or Duodenal switch: 17 with DM2 and 17 without DM2 + 17 eutrophic individuals (control group)	Pre-surgery, (before and after 3 months form dietetic intervention) and 1 year following surgery	TMAO and gamma-butyrobetaine with no increased values in obese individuals, when compared to the control group, but high after RYGB. Such changes suggest alteration in intestinal microbiota following RYGB.

SL=sleeve vertical gastrectomy; LPS=lipopolysaccharide; RYGB=Roux-en-Y bypass; TMAO=N-oxide of trimethylamine; DM2=Diabetes M*elittus* type 2

**TABLE 3:** Usage of probiotic bacteria in individuals subjected to bariatric surgery.

Name, year	Objectives	Type of study	Time of study	Sample	Time of surgery	Main results found
Chen *et al.*, 2016	To determine whether administration of probiotics improves gastrointestinal symptoms after RYGB.	Prospective randomized double-blind	March 2010 - September 2010	60 patients subjected to Gastric Bypass (mini gastric bypass and RYGB) with gastrointestinal symptoms: 20 supplemented daily with 5 billion *Clostridium butyricum* MIYAIRI; 20 supplemented with 8 billion *Bifidobacterium longum* BB536 and 20 supplemented with digestive enzymes	Individuals between 3 and 12 months post-surgery	Administration of probiotics (*Clostridium butyricum* MIYAIRI and *Bifidobacterium longum*BB536) or digestive enzymes may have their gastrointestinal symptoms reduced and a better quality of life following gastric bypass
Fernandes, 2016	To investigate the effects of prebiotic and symbiotic supplementation on inflammatory markers and anthropometric indices in subjects submitted to open RYGB.	Prospective Randomized, controlled, triple-blind	October 2013 - April 2014	18 individuals 9 subjected to RYGB and 9 healthy, divided in 3 groups: placebo (6g of maltrodextrine per day), prebiotic (6g of FOS per day) and symbiotic (6g of FOS + 1x10^**9**^ *L. paralisei + L. rhamnosus +L. acidophilus + B. lactis*), all of them supplemented for 14 days.	NI	There was no reduction of the inflammatory markers between groups after supplementation. BMI reduction and the increase of the %EWL was higher among the placebo and prebiotic groups, when compared to the symbiotic supplemented group.
Woodard, 2009	To verify whether the administration of probiotics following RYGB can influence the quality of life related to the presence of gastrointestinal symptoms, bacterial overgrowth and weight loss following surgery.	Prospective randomized controlled	From 2006 to 2007	35 morbidly obese individuals subjected to RYGB: 15 supplemented with 2.4 billion *Lactobacillus* (Puritan’sPride®) daily and 20 of the control group	Pre-surgery to 6 months	The supplement treatment with probiotics reduced the bacterial overgrowth, increased the availability of vitamin B12 and the weight loss following RYGB

RYGB= Roux-en-Y bypass; NI=not informed; %EWL=excess weight loss percentage

## RESULTS

**FIGURE 1:**
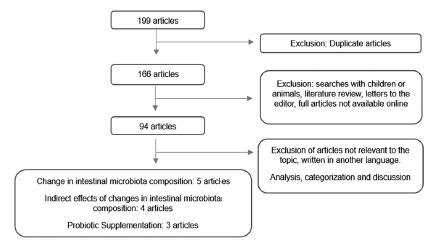
Flowchart of the article selection for the articles of the review

After the association of terms and exclusion of repeated papers in each database search, 33 articles were found in Medline; 166 in Pubmed and no paper was found by the search in Lilacs database. The 33 articles found in Medline were also indexed in the Pubmed database, thus 166 articles remained. Among these, 94 had been conducted in adults - but with an approach that did not meet the objectives of this study and, thus, did not fulfill the enrollment criteria. At the end of the search, 12 papers were selected, which were analyzed and discussed (Figure 1). 

The studies evaluating changes in the intestinal microbiota composition (Table 1) suggested that there is a relative increase of the phyla Bacteroidetes and Proteobacteria following surgery, along with a decrease of Firmicutes. It was observed that, regarding the changes in plasma levels of intestinal bacteria metabolic products (Table 2), there was a rise of trimethylamine N-oxide (TMAO); histidine; alterations in tryptophan, heme and phenylalanine metabolism; and a fall of lipopolysaccharide (LPS) and LPS binding protein, suggesting a decrease in the intestinal permeability and in the inflammatory potential in those individuals.

Regarding the studies evaluating the supplementation with probiotic bacteria alone or in tandem with prebiotic (symbiotic) (Table 3), the results indicated that supplementation with *C. butyricum* and *B. longum* reduced the gastrointestinal symptoms and improved the life quality of individuals subjected to vertical gastrectomy and that a daily ingestion of 2.4 bi *Lactobacillus* sp*.* provided better results regarding bacterial overgrowth, the availability of B12 vitamin and weight loss following RYGB. One study, however, found that a daily ingestion of 1x10^**9**^ *L. paracasei, L. rhamnosus, L acidophilus* and *B. lactis* along with 6 g of FOS for 15 days did not demonstrate superior results to that found in the placebo or prebiotic group.

## DISCUSSION

According to the findings of the present study, bariatric surgery appears to alter in a positive way the intestinal microbiota. The increase in the population of the Bacteroidetes phylum seems to be related in a negative way to corpulence, and the Firmicutes/Bacteroidetes seems to lower during the loss of weight, along with an increase of Proteobacteria. This change was observed in more minutely in the studies, mainly observing the increase of *E. coli* (belonging to the Proteobacteria phylum) and decrease of Clostridia and *Lactobacillus* (belonging to the Firmicutes phylum)^7^.

Although intestinal microbiota seems to present itself in a relatively stable way, variations between individuals may be large. Some analysis methods have identified that two phyla of bacteria, Bacteroidetes and Firmicutes, constitute more than 90% of the known dominant phylogenetic categories of the distal intestine and that, in obese individuals, there is a lower rate of Bacteroidetes, in relation to Firmicutes^2^ ^,^ ^14^ ^,^ ^15^.

The Firmicutes phylum includes more than 200 genera, many of which with a better efficiency of calorie take-up than Bacteroidetes. That occurs through the catabolism of polysaccharides from the diet, turning them into monosaccharides and short-chain fatty acids (such as butyrate, propionate and acetate). The short-chain fatty acids act in the regulation of intestinal hormones, lowering the diet ingestion, and have protective effects against insulin resistance and diet-induced obesity^2, 17^.

Also, as quoted by Bays et al.^2^, it has already been observed in rats that the process of body fat accumulation by microbiota action includes various mechanisms. Among them, stand out the increase of digestive enzymes for carbohydrates that lead to the increase of the intestinal absorption of monosaccharides; the reduction of hepatic and muscular fat oxidation; the suppression of adiposity factor secretion induced by fasting, which reduces both the oxidation of adipose tissue and the decoupling of the adenosine triphosphate generation from adipose tissue, reducing thermogenesis; increased activity of the Sterol 1 regulatory element binding protein which promotes lipogenesis; increased absorption of nutrients by increasing capillary density of vessels of the small intestine; alteration of bile acid metabolism; effects on appetite and satiety, reduction of intestinal hormones (such as glucagon-like peptide 1) and neurobehavioral brain centers.

Among the reasons for the change in intestinal colonization following bariatric surgery, changes in eating habits are of particular importance, with the reduction of fat intake and the augmentation of polysaccharide and the alteration of intestinal acidity. In the RYGB technique a small gastric pouch is made, and the distal stomach and the small intestine are excluded from the alimentary transit, anastomosing the distal end of the middle jejunum with the gastric pouch. The stomach acidity is ignored, taking the reduction of hydrochloric acid in the intestine. Studies in bacterial cultures have shown an inhibition of Bacteroidetes growth by means of pH reduction^7^. Kong *et al.* ^*10*^ have shown a significant rise of Proteobacteria related to the eating changes following surgery. Also, the presence of oxygen in the intestines seems to result from anatomic alterations which come from the surgical procedures, and favor the growth of anaerobic bacteria, such as *E. coli* ^11^.

Changes in serum levels of substances derived from the metabolism of intestinal bacteria also justify the alterations found. The study by Sarosiek *et al.* ^21^ suggests that histidine metabolites could serve as markers of metabolic changes associated with weight loss by bariatric surgery. In the same regard, LPS decrease with a subsequent inflammation reduction has been observed in patients subjected to Sleeve vertical gastrectomy, due to reduction of bacterial translocation, decreased by low fat diets^4^. However, the augmentation of TMAO, which promotes the rise in the risk of cardiovascular diseases (CVD) was unexpected, since bariatric surgery reduces CVD risk^26^.

Modesitt *et al.* ^13^ have observed that decreased conversion of tryptophan to kynurenine by inactivation of Indoleamine 2,3-dioxygenase has indicated the reduction of inflammatory cytokines in plasma and that the increased conversion of tryptophan to 3-indoxyl sulfate may reflect a change of the intestinal microbiota in these patients, since this is a metabolite of the bacterial fermentation of the amino acid. Similarly, the increase of Heme and Phenylalanine in this study has been associated to a better in the inti-inflammatory profile and a potential alteration of the intestinal bacteria.

The oral administration of bacteria beneficial to the host is being investigated. Studies indicate that the usage of probiotics prevents and treats various health disorders, such as gastrointestinal infections, inflammatory intestinal disease, lactose intolerance, urogenital infection, cystitis, fibrosis, many kinds of cancer, reduces collateral effects of antibiotic therapy, prevents dental caries, periodontal diseases and halitosis^23^.

In this compilation of studies regarding probiotic supplementation in patients subjected to bariatric surgery, it has been observed that oral administration of probiotics has reduced gastrointestinal symptoms in patients following surgery. One of the explanations for those symptoms is bacterial overgrowth, due to the presence of the “blind pouch” after RYGB³. Woodard *et al.* ^28^ have observed a decrease of bacterial overgrowth from six weeks after the usage of probiotics, staying low after three and six months afterwards, unlike the individuals in the control group. In a study by Chen *et al.* ^3^, a reduction of symptoms has been observed from as early as two weeks post-treatment with probiotics. Though different strains have been used, both studies have shown positive results in regard to the usage of probiotics and the improving of the gastrointestinal profile.

The findings of Woodard et al.^28^ have also shown a greater weight loss and an increase of serum levels of vitamin B12 via synthesis by intestinal bacteria among individuals following supplementation with probiotics. The increase of vitamin B12 synthesis by intestinal bacteria was also observed in a study by Presti *et al.* ^*19*^. Due to reduction of the absorption of vitamin B12 as a result of the decrease of the production of the intrinsic factor in gastrectomized patients, the synthesis of this vitamin by the microbiota becomes an important scientific finding, which may contribute to the reduction of the nutritional deficiencies in this population.

In the other hand, the study by Fernandes *et al.* ^*6*^ found no association between the inflammatory markers, as well as anthropometric indexes, and the usage of symbiotics. However, the study has its limitations, such as the usage of the prebiotics in tandem with the probiotics; a sample too little; and a small window of intervention time, and as such more studies are needed as to confirm this result. It is worth noting, nevertheless, that Furet et al.^7^ found a significant relationship between *Faecalibacterium prauzitzii, E. coli*, and Bacteroidetes*/Prevotella*, following surgery, and the reduction of low-grade inflammation associated with obesity, what indicates the action potential of microorganisms in the inflammatory parameters.

The studies regarding the usage of probiotics in relation to bariatric surgery are unfortunately in small number. Even so, the results found hitherto are promising and suggest significant benefits to the population subjected to surgery. Thus far few strands have been used in the studies and not many an information has been published regarding the starting time for probiotic administration and the duration of the treatment until the remission of gastrointestinal symptoms and the synthesis of vitamins. Other benefits form the usage of probiotics, such as reduction of lactose intolerance; better digestion of proteins; and the increase of vitamin and mineral bioavailability^18^ have already been identified in other researches and should be investigated in this population, lest the impact of intestinal bacteria in the health of individuals subjected to bariatric surgery may be thoroughly understood.

## CONCLUSION

Bariatric surgery favors changes in intestinal microbiota, with a relative increase of the Bacteroidetes and Proteobacteria phyla, along with a reduction in Firmicutes. This is possibly due to changes in the gastro-intestinal flux, coupled with a reduction in acidity, in addition to changes in eating habits. The usage of probiotics seems to reduce gastrointestinal symptoms in the post-surgery, favor the increase of vitamin B12 synthesis and potentiate weight loss. Unfortunately, studies in the field are scant, and more clinical research is needed as to reiterate the results that have hitherto been found, and as to verify the influence of probiotic supplementation in the quality of life, in the alimentary intolerances and in the metabolic, as well as in the inflammatory, profile of this population, since such factors are so significantly changed following bariatric surgery.
